# Assessment of Gynecologic Emergency Triage and Appointment Wait Times: A Mystery Caller Study

**DOI:** 10.7759/cureus.92541

**Published:** 2025-09-17

**Authors:** Melanie G Mandell, Brisa Avila, Kori A Baker, Kimberly Christnacht, Rebekah Coelho, Tamara Corley, Douglas Fritz, Kristina E Johnson, Neya Manavalan, Madeleine M Mason, Payton Moody, Tyler Muffly

**Affiliations:** 1 Obstetrics and Gynecology, University of Colorado School of Medicine, Aurora, USA; 2 Obstetrics and Gynecology, Denver Health Medical Center and Hospital, Denver, USA

**Keywords:** access to health care, gynecologic emergency, health care staff, mystery caller study, triage

## Abstract

Background: Front desk staff are often responsible for triaging patients, despite lacking formal medical training. Incorrect triage recommendations could result in patients delaying care for emergent conditions. No research exists to characterize triage accuracy in the gynecologic outpatient setting.

Objective: We investigated the ability of clinician office staff to identify emergent conditions and advise patients accordingly. Additionally, we sought to understand the influence of private versus public insurance on appointment wait time and triage recommendations.

Study design: Obstetrician-gynecologist (OB/GYN) office phone numbers were identified from the Centers for Medicare and Medicaid Services National Practice and Provider Enumeration System and contacted using a covert mystery caller study approach. Half of the calls described a gynecologic emergency that required immediate intervention, while the other half described a gynecologic condition that required urgent evaluation. In the “Emergent” scenarios, callers described a positive pregnancy test after tubal ligation or a tubo-ovarian abscess. In the “Urgent” scenarios, callers described a urinary tract infection or recurrent vaginitis. The date of the soonest appointment and triage recommendations were recorded.

Results: Our sample included 1,114 calls to 557 unique physician offices from 48 states (excluding North Dakota and Rhode Island). Of the physician offices contacted, 371 physicians met the inclusion criteria. In this sample, 75.2% of OB/GYN offices accepted Medicaid. The median physician age was 53 (IQR 44-61), and almost two-thirds (63.2%) of physicians contacted were female. A total of 142 callers were instructed to seek emergency care, transferred to a nurse triage line, or offered provider consultation. Of Emergent scenario callers, 21.5% (n = 60/N = 279) were correctly triaged. Eighty-two of the 279 Urgent scenario callers (29.4%) were incorrectly instructed to seek emergency care. There was no significant difference in appointment wait time or triage recommendation based on insurance. Calls to female physicians were associated with shorter wait times (p < 0.01), while academic physicians (p < 0.01) and longer call hold times (p = 0.04) were associated with longer wait times. Wait times varied significantly between clinical scenarios.

Conclusions: To the best of our knowledge, this is the first nationwide audit of OB/GYN office staff patient triage. Our results revealed that patients are often incorrectly triaged and offered future appointments for emergency conditions that require immediate attention. Faulty triage results in significantly delayed treatment, which can impede patients’ future fertility or threaten their lives. We propose the development of a standardized resource to increase telephone triage accuracy and minimize patient harm.

## Introduction

Patients with time-sensitive gynecologic concerns often experience significant discomfort before treatment [1]. Beyond the physical discomfort of these conditions, they also disrupt patient livelihood, decrease self-esteem, and contribute to interpersonal relationship stress [2]. One study found that over 59% of the cost of urinary tract infections treated with antibiotics is due to indirect losses, such as missed work and inability to complete unpaid labor [3].

Given the acuity and pervasiveness of gynecologic symptoms, many patients understandably desire immediate relief. However, the time from requesting an appointment to being seen by a physician varies significantly based on clinic resources and location [4], and long waits also have a negative psychological impact on patients [5]. Acute gynecological concerns may also be emergencies that require immediate medical attention, including pelvic inflammatory disease, ectopic pregnancy, and pyelonephritis [6]. When patients call the clinic to schedule appointments for their concerns, they ideally will encounter office staff who can identify the signs and symptoms of an emergency over the phone and advise them accordingly. However, the American College of Obstetricians and Gynecologists (ACOG) does not currently provide guidelines to screen for medical emergencies over the phone [7]. Thus, patients with conditions requiring emergency care may not be appropriately directed to the emergency department (ED) if the intake personnel cannot correctly identify symptoms.

Long wait times and incorrect emergency recommendations may be exacerbated by insurance type. Previous studies have demonstrated increased wait times for patients with public insurance seeking obstetrics and gynecologic (OB/GYN) subspecialty care [8]. We hypothesized that patients with Medicaid insurance would face longer appointment wait times when seeking care for an acute gynecologic concern than patients with private payer insurance. We also hypothesized that office staff would overlook most gynecologic emergencies and schedule patients for appointments instead of escalating their care by recommending the patient visit the ED, transferring them to a nurse triage line, or offering provider consultation.

## Materials and methods

Informed consent

The mystery caller study received approval from the Colorado Multiple Institutional Review Board (#23-1303), which explicitly exempted participating offices from obtaining informed consent. However, a debriefing letter was dispatched to the participating practice after each call. The debriefing letter informs participants about their participation in a mystery caller study evaluating front desk staff triage and appointment wait times for OB/GYN patients with public versus private insurance.

Study participants and identification of practices

A list of physicians was accessed from the Centers for Medicare and Medicaid Services National Practice and Provider Enumeration System. Providers were removed from the list if they were retired, practicing outside of the United States, or lacked an active board certification. Finally, the data were limited to providers with “obstetrics and gynecology” listed as their taxonomy code. Physician demographic information was compiled from healthgrades.com, a patient-facing database, and physicians were categorized by age and geographical region. One physician per office was called to minimize administrative burden, and duplicate phone numbers and addresses were removed. Only general OB/GYN offices were called. An OB/GYN was excluded from the study if the number contacted did not correspond to the expected OB/GYN (i.e., an academic office), a physician referral was required before scheduling, medical records were required before scheduling, the phone number was a physician’s personal phone, the call went to voicemail, there was no answer or a busy signal on repeat calls, greater than five minutes were spent on hold, the practice was part of a closed health system (e.g., Kaiser, military hospital), or the physician was not accepting new patients. If the caller was on hold for more than five minutes, the call was marked “unanswered” and recalled 24 hours later. If a call was not answered on the first attempt, a second call was attempted 24 hours later before the office was marked “unreachable.” Callers specifically asked to see the OB/GYN physician of interest.

Study design

Ten trained investigators called each of the 703 randomly assigned physicians before starting data collection to confirm that the physicians were currently practicing (Appendix A). After initial screening, 557 physicians were identified for inclusion. The investigators used a standardized script for four scenarios, two “Emergent” and two “Urgent” conditions, to evaluate triage accuracy and appointment wait times (Appendix B). The “Emergent” scenarios described a positive pregnancy test after a tubal ligation or a previously diagnosed 6-cm tubo-ovarian abscess with new green discharge. The positive pregnancy test after a tubal ligation was intended to raise suspicion for an ectopic pregnancy [9]. A large tubo-ovarian abscess with increasing pain and green mucopurulent cervical discharge indicates sub-optimal antibiotic treatment and requires surgical management [10]. The “Urgent” scenarios described recurrent vaginitis or acute cystitis. All initial questions were directed at the answering receptionist.

Two calls were made to each physician's office using the same scripted clinical vignette. In the first call, the caller claimed to possess Blue Cross/Blue Shield (BCBS), a popular private insurance. In the other call, the caller claimed to possess Medicaid insurance. The caller's identity and the order in which the calls were made (BCBS vs. Medicaid) were randomized. Each clinical vignette was assigned to an equal number of physician offices.

The phone calls were conducted from 8 am to 5 pm (excluding the 12-1 pm lunch break), local time, for six business days, from Monday, July 15th to Monday, July 22nd, 2024. We intended for the calls to occur over one week. However, a national Microsoft outage on Friday, July 19, 2024, affected the ability of physician offices to schedule patients and access electronic health records. Therefore, the calling window was expanded to include one additional business day. Calls were placed during this brief window to decrease the potential for errors arising from prolonged intervals between calls.

Data collection

Callers recorded the earliest appointment date, total call time, number of transfers, insurance acceptance, and whether the patient received an ED referral. An ED referral was defined as either (1) a recommendation to bypass the OB/GYN office and go to the ED to seek a higher level of care, (2) the staff member transferring the patient to nurse triage, or (3) offering provider consultation. Providing a same-day appointment did not count as an ED referral. At the end of the call, each caller clarified that no appointment was made so that the study would not prevent actual patients from accessing care.

Call report forms were collected and managed using REDCap (Research Electronic Data Capture; Vanderbilt University, Nashville, TN, USA) hosted at the Denver Health Office of Research [11,12].

Data analysis

Time to the earliest appointment was quantified as the number of business days from the initial call. A generalized linear mixed Poisson model with a log-link was employed. In this model, physicians were considered random effects with repeated measures, as each physician received up to two calls. Additionally, we included insurance type as a fixed effect while controlling for physician gender, age group, ACOG District, day of the week, and physician medical degree. We created a second model that introduced an interaction term between case urgency and insurance type to better understand the impact of insurance type. All analyses were conducted in R 4.4.1 (www.r-project.org; The R Foundation for Statistical Computing, Vienna, Austria). Models were created using the R package "lme4," and estimated marginal means were calculated using the "emmeans" package.

Statistical power

An a priori power analysis identified the required sample sizes for both primary and secondary outcomes. To detect a 10% difference in ED referral rates with 80% power, we needed 584 total participants. For detecting a small difference in appointment wait times, a total of 788 participants was required. These estimates guided recruitment targets and ensured the study was adequately powered to detect effects of clinical and policy relevance.

## Results

Appointment accessibility

**Figure 1:**
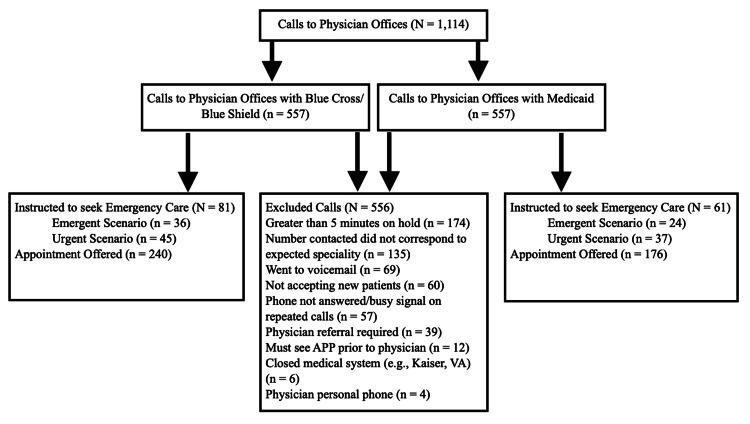
Schematic representation of phone calls placed to physician offices during data collection. APP: advanced practice provider

Overall, 1,114 individual phone calls were completed to 557 unique physicians in 48 states plus the District of Columbia. No calls were made to North Dakota or Rhode Island despite the randomization. Of the 1,114 phone calls, 88.6% (n = 988) successfully reached a front desk representative, while 11.3% of calls (n = 126) did not yield a connection despite two separate attempts. Among the 126 unsuccessful connections, 54.7% (n = 69) were redirected to voicemail, and 45.2% (n = 57) reached a busy signal. Of the remaining 988 successful connections, 556 were excluded (Figure 1).

Ultimately, 371 of the 557 physicians met the inclusion criteria. Of these 371 physicians, 342 (92.2%) accepted Medicaid, while 371 (100%) accepted BCBS. A total of 302 physicians accepted Medicaid and/or BCBS and gave a date for the soonest available appointment. One hundred and twenty calls (32.3%) resulted in the patient being referred to the ED.

Physician characteristics

Physician characteristics are reported in Table 1.

**Table 1:** Demographics of physicians contacted, including those that did not meet inclusion criteria (N = 557). * Only lists district territories within the 50 states of the United States of America and the District of Columbia.

Characteristics	N (%)
Age (Years)	
Less than 50 years old	214 (39.9)
50 to 55 years old	86 (16.0)
56 to 60 years old	85 (15.8)
61 to 65 years old	67 (12.5)
Greater than 65 years old	85 (15.8)
Gender	
Female	353 (63.4)
Male	204 (36.6)
Medical School Training	
Allopathic training	513 (93.8)
Osteopathic training	34 (6.2)
Medical School Location	
US senior medical student	408 (82.1)
International medical graduate	89 (17.9)
Academic Affiliation	
Private practice	500 (89.8)
University	57 (10.2)
Geographic Classification	
Metropolitan area	507 (91.0)
Rural area	50 (9.0)
Number of Phone Transfers	
No transfers	352 (63.2)
One transfer	158 (28.4)
Two transfers	37 (6.6)
More than two transfers	10 (1.8)
Age Category	
Less than 40 years old	76 (14.2)
40 to 49 years old	138 (25.7)
50 to 59 years old	171 (31.8)
60 to 69 years old	118 (22.0)
70 years and older	34 (6.3)
American College of OBGYNs Districts*	
District I (CT, ME, MA, NH, RI, VT)	34 (6.1)
District II (NY)	33 (5.9)
District III (DE, NJ, PA)	37 (6.6)
District IV (DC, GA, MD, NC, SC, VA, WV)	65 (11.7)
District V (IN, KY, OH, MI)	54 (9.7)
District VI (IL, IA, MN, NE, ND, SD, WI)	64 (11.5)
District VII (AL, AR, KS, LA, MS, MO, OK, TN)	93 (16.7)
District VIII (AK, AZ, CO, HI, ID, MT, NV, NM, UT, WA)	93 (16.7)
District IX (CA)	30 (5.4)
District XI (TX)	20 (3.6)
District XII (FL)	34 (6.1)
Scenario	
Acute cystitis (“Urgent”)	136 (24.4)
Recurrent vaginitis (“Urgent”)	141 (25.3)
Positive pregnancy test after a tubal ligation (“Emergent”)	139 (25.0)
Prior trip to ED and was found to have a 6 cm TOA (“Emergent”)	141 (25.3)
Central Scheduling	
Yes, central scheduling number	201 (36.1)
No	356 (63.9)
Day of the Week Called	
Monday	58 (10.4)
Tuesday	112 (20.1)
Wednesday	172 (30.9)
Thursday	132 (23.7)
Friday	83 (14.9)

Emergent scenario ED referrals

**Figure 2:**
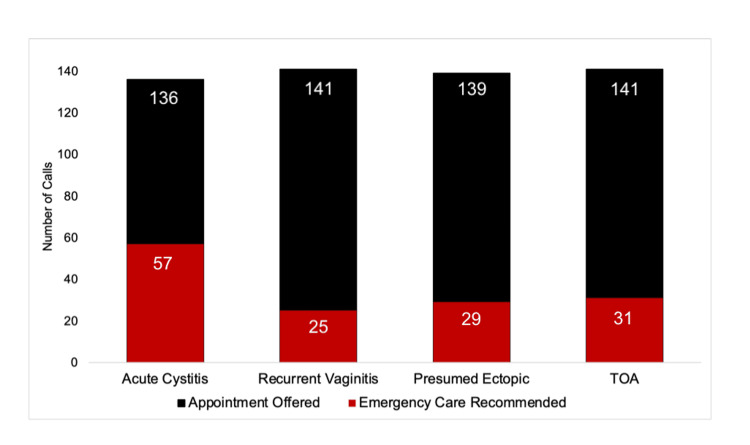
Triage recommendations by front desk staff for urgent and emergent gynecologic cases. Acute cystitis, an “Urgent” scenario, received the most emergency department referrals out of the four scenarios. Positive pregnancy test after tubal ligation, which is an assumed ectopic pregnancy, and TOA should have received all emergency department referrals from office staff. TOA: tubo-ovarian abscess

Of the 279 Emergent scenario calls that should have received ED referral instructions, only 21.5% (n = 60) were appropriately triaged to emergency care. Thirty-one of the 135 tubo-ovarian abscess calls received ED referrals (23.0%), while 29 of the 142 potential ectopic pregnancies (positive pregnancy test after tubal ligation) received ED referrals (20.4%) (Figure 2).

Urgent scenario ED referrals

Of the 279 Urgent scenario calls, 29.4% (n = 82) were incorrectly triaged to a higher level of care. Patients presenting with acute cystitis received 57 referrals to the ED, while recurrent vaginitis patients received 25 referrals (Figure 2).

ED referral patterns

For Urgent scenarios, BCBS beneficiaries were 1.28 times more likely than Medicaid patients to be referred to the ED (18.4% vs. 14.4%, p = 0.25). For Emergent scenarios, BCBS beneficiaries were 1.25 times more likely than Medicaid patients to be referred to the ED (13.6% vs. 11.1%, p = 0.4405).

Among all ED referrals, 97 cases (adjusted rate 68.3%) were from immediate front desk staff referrals, 32 cases from nurse triage (adjusted rate 22.5%), and 13 (adjusted rate 9.2%) from provider consultation. The model showed significant physician-level random effects (variance = 0.42, p < 0.001), indicating substantial variation in referral practices between individual providers.

Wait time analysis by scenario and insurance type

The median wait time across all scenarios was eight business days (IQR 0-26 days). For patients presenting with tubo-ovarian abscess, BCBS beneficiaries waited a median of 4.8 days (95% confidence interval (CI): 3.2-7.2), while Medicaid recipients experienced 37.5% longer waits at 6.6 days (95% CI: 4.4-9.8, p < 0.01). In cases of suspected ectopic pregnancy after tubal ligation, BCBS patients waited 6.4 days (95% CI: 4.3-9.5), while Medicaid patients had 12.5% shorter waits at 5.6 days (95% CI: 3.8-8.3, p < 0.01).

Patients with urinary tract infections had the shortest wait times, with BCBS patients waiting 3.2 days (95% CI: 2.1-4.9) and Medicaid patients experiencing 9.4% shorter waits at 2.9 days (95% CI: 1.9-4.4, p < 0.01). For vaginitis cases, BCBS patients waited 8.3 days (95% CI: 5.7-12.1), and Medicaid patients experienced 18.1% shorter waits at 6.8 days (95% CI: 4.6-10.0, p < 0.01).

Multivariate predictors of wait time

After adjusting for physician-level clustering and other covariates, patients seen in academic practice settings experienced 12% longer wait times (incidence rate ratio (IRR) = 1.12, 95% CI: 1.08-1.16, p < 0.01). For each additional minute of hold time, wait times increased by 5% (IRR = 1.05 per minute, 95% CI: 1.02-1.08, p = 0.04). Urgent scenarios had 9% longer wait times than emergent ones (IRR = 1.09, 95% CI: 1.04-1.14, p < 0.01). Insurance type was not significantly independently associated with wait times after accounting for other factors. The random effects component revealed significant physician-level variation, with a variance of 3.59 and an intraclass correlation coefficient of 0.98.

## Discussion

Principal findings

Emergency gynecological conditions were correctly identified and responded to by front desk staff in a minority of cases. Among the 279 calls describing an Emergent scenario, only 60 (21.5%) were correctly identified as emergencies and directed to emergency care, transferred to a nurse triage line, or offered provider consultation. A secondary finding of our study was that appointment wait times were not independently associated with insurance type, contradicting our hypothesis that patients with Medicaid insurance would face significantly longer wait times and emphasizing the dominant role of clinical and practice-related factors. However, when stratified by scenario type, Medicaid beneficiaries experienced significantly shorter wait times in three of the four scenarios. While our mystery caller design ensured that these simulated patients were not at risk, the results raise serious concerns that actual patients facing life-threatening emergencies may be similarly mismanaged when they contact their OB/GYN offices.

Results in the context of what is known

The observed triage deficiency aligns with findings from a similar study of musculoskeletal urgent care centers, where front desk staff referred less than 20% of callers with symptoms of lower extremity compartment syndrome - a limb-threatening condition - to the ED [13]. Interestingly, less than 5% of callers received additional triage questions, and those who did were four times as likely to be instructed to seek emergency care [13], indicating that this is a potential area for improvement in clinics.

Previous OB/GYN mystery caller studies that analyzed appointment wait time by insurance type have noted better access to care for patients with private insurance when seeking subspecialist treatment [8] and no significant difference when seeking care from OB/GYN generalists [14]. In keeping with the literature, our study found no significant difference in wait time by insurance for appointments with OB/GYN generalists when calls were analyzed in aggregate.

Clinical implications

Delays in seeing an OB/GYN may result in missed opportunities for early detection and management of conditions such as sexually transmitted infections or extrauterine pregnancies, potentially leading to more advanced disease and costly treatment. To address this, interventions should focus on triage accuracy and systemic barriers to care. Telehealth may be able to alleviate some of the existing healthcare disparities. Previous research has demonstrated that gynecologic telehealth visits are cost-effective and have patient satisfaction scores comparable to traditional in-person visits [15]. The expansion of telehealth utilization may decrease wait times and improve access to gynecologists without impacting the quality of care delivered. However, Medicaid reimbursement for telehealth services varies by state, which may hinder widespread adoption [16]. Policy changes to standardize and increase Medicaid reimbursement for telehealth could reduce financial barriers for providers and improve care delivery in underserved populations.

Another promising approach involves transparency in wait times. The Veterans Administration, for example, provides daily wait time updates online [17], allowing patients to make informed decisions about where to seek care. Expanding such dashboards to other healthcare systems could empower patients while incentivizing clinics to reduce delays.

Research implications

Instructing a patient to seek emergency care or transferring telephone patients to a nurse or clinician rests on the non-clinical front desk staff. However, these staff members often lack formal medical training. Our analysis revealed significant variation in emergency referral practices between physician offices; therefore, we believe OB/GYN office staff would benefit from standardized front desk staff training to identify patient emergencies. A brief literature search revealed that the University of Bern implemented training for fifth-year medical students to help them identify emergencies over the phone [18]. Medical students reported learning valuable skills after only an afternoon and a desire for more similar training courses. At present, no such resource exists for OB/GYN offices. The Society of Urologic Nurses and Associates developed a guidebook for their nurses that provides clear instructions for patient referral based on keywords and timing of symptoms [19]. Development of a standardized workflow and training in identifying medical emergencies, specifically in OB/GYN generalist offices, could improve patient outcomes.

Our study did not track which patients were asked for more details about their symptoms, nor did we instruct callers to remain on the line to speak with triage nurses when offered out of respect for the caller and staff member time. In the future, these variables should be measured to better characterize the efficacy of triage questions in patient management.

Limitations

While our study revealed a statistically significant difference in wait times between urgent and emergent clinical vignettes, several important limitations must be acknowledged. First, our "mystery patients" were standardized to have either commercial insurance (BCBS) or Medicaid. As a result, these findings may not be generalizable to patients with other types of insurance or those without insurance coverage. Second, our methodology may underestimate the real-world difficulties of identifying and accessing care, especially for vulnerable populations. Lastly, while efforts were made to minimize sampling bias, such as employing a stratified national sampling approach, not all states were represented or equally represented, and the potential for unmeasured confounding variables remains. Future research should further refine these methodologies, explore additional patient insurance scenarios, and incorporate more diverse clinical settings to enhance the generalizability of findings.

Strengths

Despite these limitations, our study has several notable strengths. Like prior mystery caller protocols, this investigation leverages a robust, nationally representative dataset stratified by geographic distribution, enhancing its applicability across diverse gynecologic care settings. The study further highlights the utility of audit-style investigations, a methodological approach that provides valuable insights into patient access and healthcare delivery while minimizing potential biases associated with self-reported or observational data. This approach closely simulates the real-world experience of patients seeking care, capturing key barriers such as insurance type and clinical urgency in a controlled and reproducible manner. Furthermore, using trained callers ensures a conservative estimate of real-world challenges, as baseline health literacy and educational differences between our callers and typical patients may exacerbate the obstacles actual patients face. Together, these strengths underscore the value of our findings and their potential to inform targeted interventions to improve patient access to timely care.

## Conclusions

To the best of our knowledge, this is the first nationwide audit of OB/GYN office staff patient triage. Our results revealed that patients are often incorrectly triaged and offered future appointments for emergency conditions that require immediate attention. Faulty triage results in significantly delayed treatment, which can impede patients’ future fertility or threaten their lives. We propose the development of a standardized resource to increase telephone triage accuracy and minimize patient harm.

## Figures and Tables

**Figure 3:**
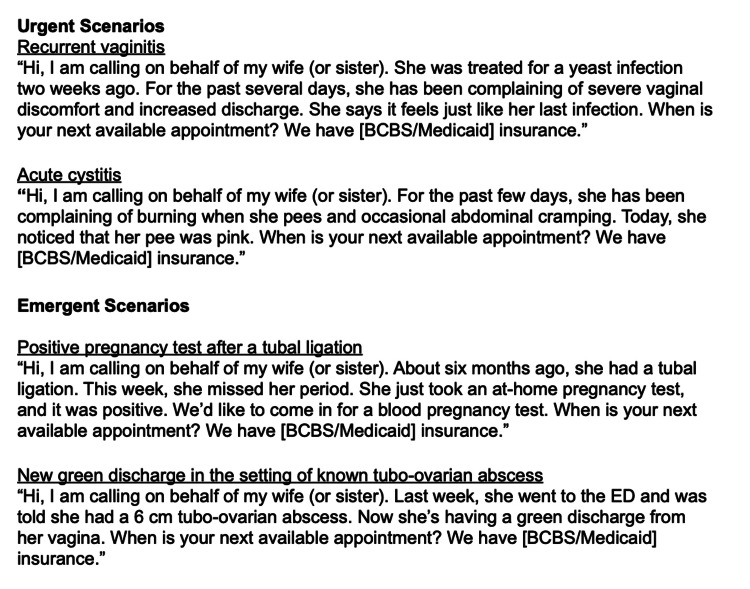
Clinical vignettes and call scripts used in data collection.

## Appendices

Appendix A 

**Table 2:** Opening script for initial screening calls to general obstetric and gynecologic offices.

“Hi! I just moved to the area and heard great things about Doctor X. Do you know when your first appointment with Doctor X might be?”

Appendix B
